# Dual PI3K/mTOR inhibitor BEZ235 as a promising therapeutic strategy against paclitaxel-resistant gastric cancer via targeting PI3K/Akt/mTOR pathway

**DOI:** 10.1038/s41419-017-0132-2

**Published:** 2018-01-26

**Authors:** Dongshao Chen, Xiaoting Lin, Cheng Zhang, Zhentao Liu, Zuhua Chen, Zhongwu Li, Jingyuan Wang, Beifang Li, Yanting Hu, Bin Dong, Lin Shen, Jiafu Ji, Jing Gao, Xiaotian Zhang

**Affiliations:** 1 Department of Gastrointestinal Oncology, Key Laboratory of Carcinogenesis and Translational Research (Ministry of Education/Beijing), Peking University Cancer Hospital and Institute, Beijing, 100142 China; 2 Department of Pathology, Key Laboratory of Carcinogenesis and Translational Research (Ministry of Education/Beijing), Peking University Cancer Hospital and Institute, Beijing, 100142 China; 3 Department of Surgery, Key Laboratory of Carcinogenesis and Translational Research (Ministry of Education/Beijing), Peking University Cancer Hospital and Institute, Beijing, 100142 China

## Abstract

Paclitaxel (PTX) is widely used in the front-line chemotherapy for gastric cancer (GC), but resistance limits its use. Due to the lack of proper models, mechanisms underlying PTX resistance in GC were not well studied. Using established PTX-resistant GC cell sublines HGC-27R, we for the first time integrated biological traits and molecular mechanisms of PTX resistance in GC. Data revealed that PTX-resistant GC cells were characterized by microtubular disorders, an EMT phenotype, reduced responses to antimitotic drugs, and resistance to apoptosis (marked by upregulated β-tubulin III, vimentin, attenuated changes in G_2_/M molecules or pro-apoptotic factors in response to antimitotic drugs or apoptotic inducers, respectively). Activation of the phosphoinositide 3-kinase, the serine/threonine kinase Akt and mammalian target of rapamycin (PI3K/Akt/mTOR) and mitogen-activated protein kinase (MAPK) pathways were also observed, which might be the reason for above phenotypic alternations. In vitro data suggested that targeting these pathways were sufficient to elicit antitumor responses in PTX-resistant GC, in which the dual PI3K/mTOR inhibitor BEZ235 displayed higher therapeutic efficiency than the mTOR inhibitor everolimus or the MEK inhibitor AZD6244. Antitumor effects of BEZ235 were also confirmed in mice bearing HGC-27R tumors. Thus, these data suggest that PI3K/Akt/mTOR and MAPK pathway inhibition, especially PI3K/mTOR dual blockade, might be a promising therapeutic strategy against PTX-resistant GC.

## Introduction

In China, >80% of gastric cancer (GC) patients are diagnosed at an advanced stage and are denied radical surgery and some chemotherapies^1^. As a microtubule stabilizer with good tolerability, paclitaxel (PTX) is widely used to treat advanced GC, yet its therapeutic efficiency is limited by acquired resistance and the following treatment failure. Thus, a better understanding of the mechanisms underlying PTX resistance is crucial for improving patient prognosis.

Our previous work verified the feasibility of PTX plus capecitabine as a first-line or second-line therapy for advanced GC, and we documented its efficacy and safety^2,3^. However, understanding how to optimize efficacy of PTX-based treatments against GC remains unclear. Developing promising biomarkers to select patients who are likely to benefit from PTX is the best strategy. We have identified biomarkers to predict poor response to PTX-containing therapies for GC, such as overexpression of β-tubulin III alone or in combination with microtubule-associated protein-tau in biopsy samples, as well as high β-tubulin III or Alpha-1-Microglobulin/Bikunin Precursor (AMBP) in serum^4–8^. Although the predictive value for primary resistance of PTX has been confirmed, whether these markers contribute to acquired resistance to PTX for GC remains unclear.

Acquired resistance is another major obstacle for improving PTX response in GC. Overexpressed β-tubulin III has been reported in numerous types of acquired PTX-resistant cancers^9–12^, but whether it is an unfavorable factor in GC is uncertain. According to published reports, acquired resistance to 1PTX in cancers involves other mechanisms, including altered drug efflux, epithelial–mesenchymal transition (EMT), microtubule dynamics, cyclin-dependent kinase 1 (CDK1), and cell death signaling^12–14^. We previously cooperated with Yashiro’s group to establish acquired PTX-resistant GC cell lines and to preliminarily link PTX resistance to enhanced drug efflux and apoptotic resistance^15^. Other researchers have reported increased expression of genes that reinforce G_2_/M transition in acquired PTX-resistant GC cells^16^. However, according to our knowledge, acquired PTX-resistant GC models have not yet been reported to integrate the underlying biological phenotypes and molecular alterations so far.

Therapeutic options for acquired PTX-resistant cancers have been developed, and these include targeting drug efflux^17^, CDK1 inhibitors^14^, stabilizing microtubules^18^, and epigenetic therapy to induce apoptosis and suppress angiogenesis^19^. Unfortunately, only fibroblast growth factor receptor inhibitor has been used to treat PTX-resistant GC^20^. Thus, more strategies for PTX-resistant GC are in urgent need. The involvement of the PI3K/Akt/mTOR or MAPK pathways has been noted in PTX resistance, and both pathways are activated in PTX-resistant prostate cancer cells compared with parental cells^21^. mTOR suppression produces antitumor effects in PTX-resistant lung cancer by inhibiting P-glycoprotein functions^22^. In GC, an Akt inhibitor has been applied to overcome primary resistance to PTX in a PTEN loss patient-derived xenograft^23^. Taken together, therapies for GC with acquired resistance to PTX are poorly understood, and targeting PI3K/Akt or MAPK signaling seems to be a promising option.

Therefore, we attempted to characterize biological and molecular hallmarks of newly established PTX-resistant GC cells and with bioinformatic screening, we also evaluated whether targeting the PI3K/Akt/mTOR or MAPK pathways exerted antitumor actions against PTX-resistant GC. Our work sheds a light upon the improvement of GC chemotherapy and provides evidence for further clinical investigation.

## Results

### Altered morphology, microtubular disorders, and EMT changes in PTX-resistant GC cells

**Fig. 1:**
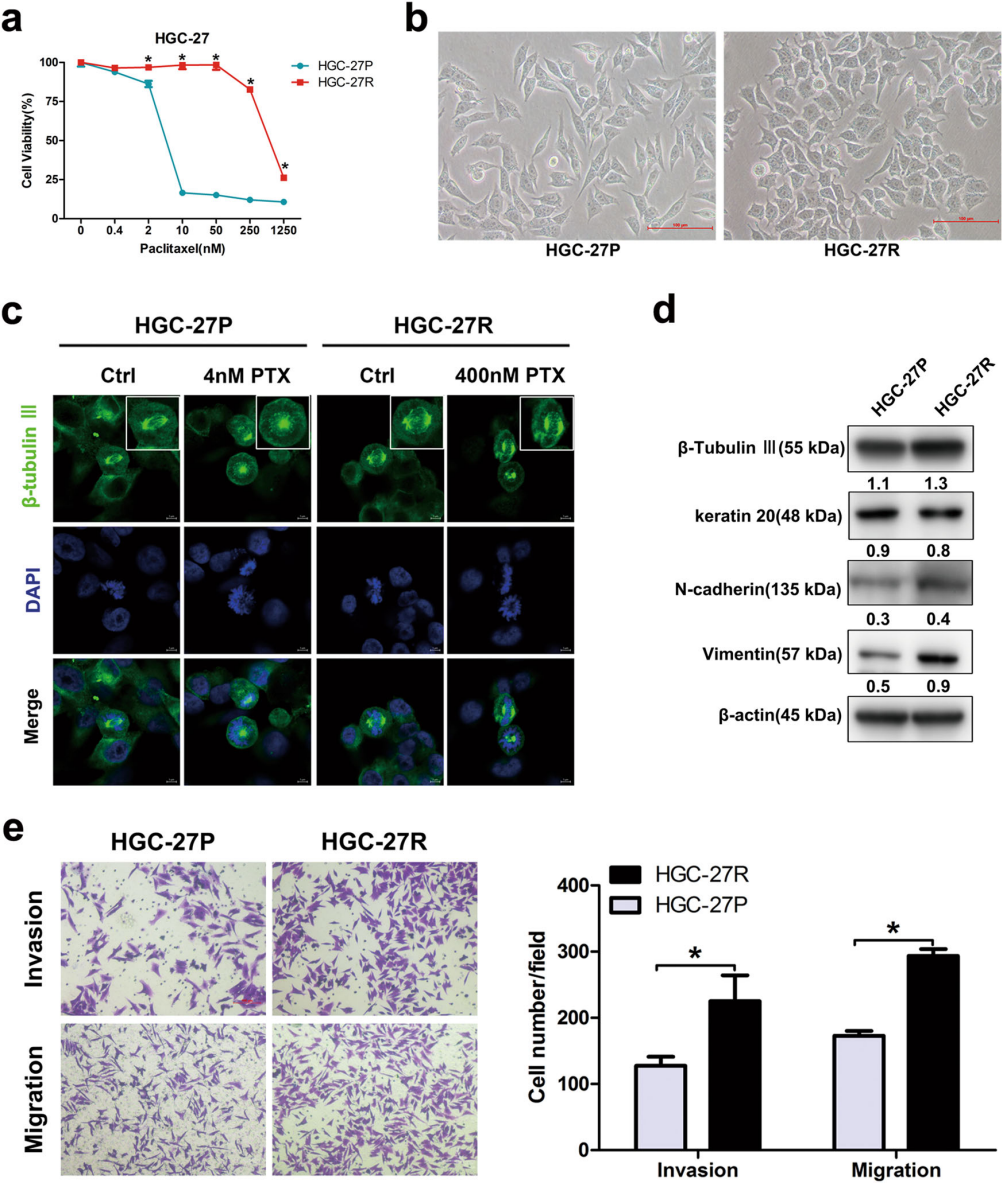
Altered morphology, microtubular disorders, and EMT in PTX-resistant GC cells. **a** Cell viability of HGC-27P and HGC-27R cells after exposure to PTX for 48 h. **b** Representative morphology of parental and PTX-resistant GC cells. Original magnification: ×200. **c** Microtubules at metaphase visualized with immunofluorescence after an exposure to PTX for 12 h. β-Tubulin III stained for microtubules and DAPI stained for nuclei (green and blue, respectively). Scale bar, 5 μm. **d** β-Tubulin III and EMT protein expression measured using western blot. **e** Invasion and migration capacity of HGC-27P and HGC-27R cells measured by Transwell assay with/without Matrigel. Data expressed as mean ± S.D. of three independent experiments. *p < 0.05

PTX-resistant cell sublines HGC-27R were established using exposure to escalating doses of PTX. Figure 1a shows half-maximal inhibitory concentration (IC_50_) values. We then confirmed altered cellular morphology after PTX resistance by microscopy assessment. HGC-27P cells were elongated with a clear boundary and HGC-27R cells were polygonal and clustered (Fig. 1b). Next, we assessed microtubule changes using β-tubulin III during metaphase when microtubules were attached to centrosomes and kinetochores. In parental cells, microtubules appeared as lines smoothly emitted from spindle pores and were aligned in an orderly fashion on the metaphase plate. Dot-like structures occurred in untreated and PTX-treated resistant cells (Fig. 1c). β-Tubulin III protein expression was upregulated in PTX-resistant cells compared with parental cells (Fig. 1d), indicating that PTX-resistant GC cells obtained features of microtubular disorders. On the other hand, the epithelial protein keratin 20 decreased but mesenchymal markers involving N-cadherin and vimentin increased in PTX-resistant cells compared with parental progenitors (particularly, vimentin; Fig. 1d), whereas PTX-resistant cells also displayed greater capacity of invasion and migration than progenitors (Fig. 1e). These results indicated that acquired morphological changes in PTX-resistant GC cells were caused by enhanced microtubular disorders and EMT process.

### PTX-resistant GC cells was less sensitive to antimitotic agents

**Fig. 2:**
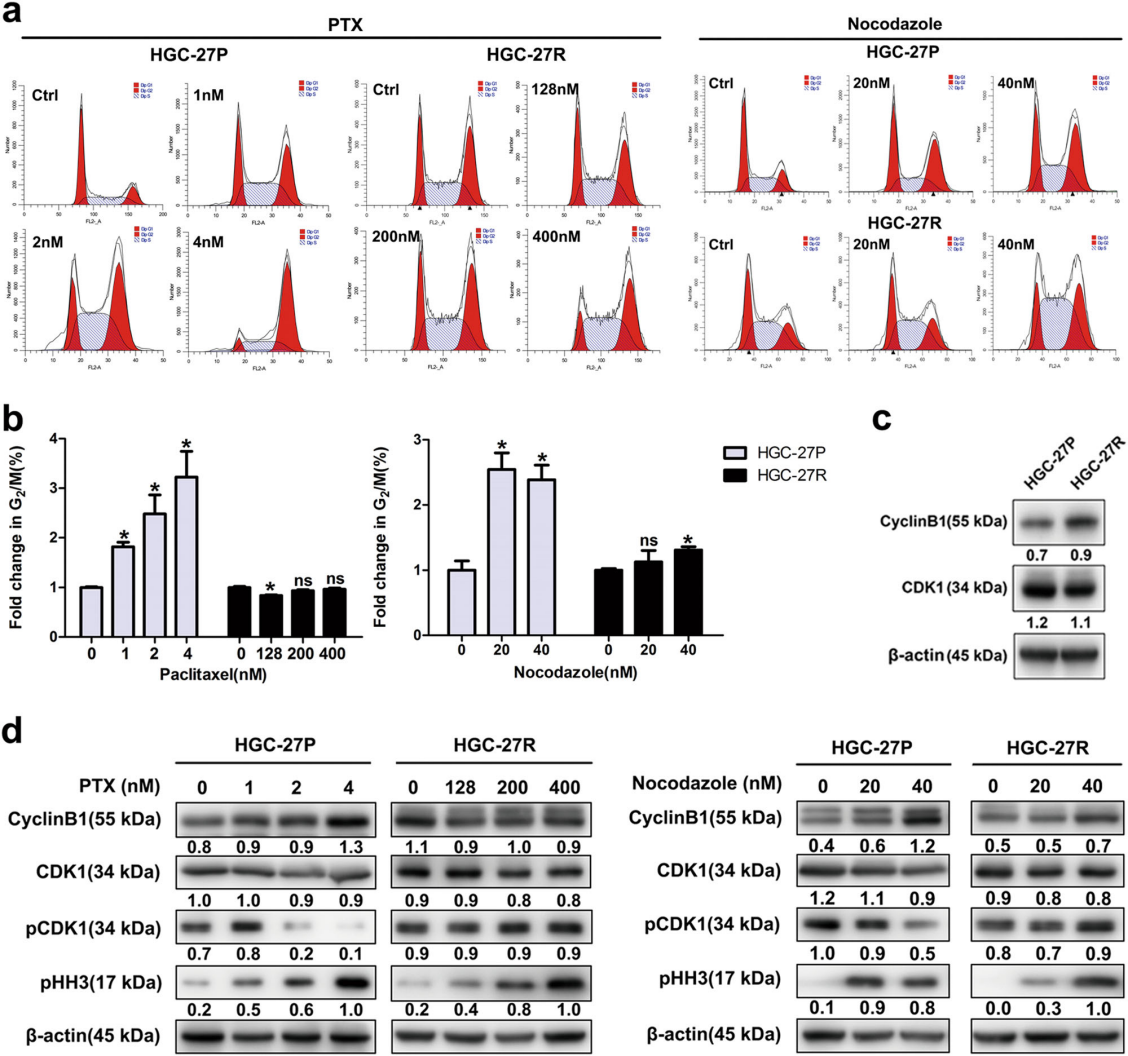
PTX-resistant GC cells was less sensitive to antimitotic agents. **a**, **b** Cells accumulated at the G_2_/M phase after exposure to PTX and nocodazole as indicated for 24 h measured by flow cytometry. **c**, **d** Cell cycle protein expression quantified with western blot. Data expressed as mean ± S.D. of three independent experiments. *p < 0.05

Induction of cell cycle arrest at the G_2_/M phase is the most prominent consequence of microtubular stabilization by PTX^24^. The two cells’ responses to antimitotic drugs (PTX and nocodazole) were compared. Unlike parental counterparts, PTX treatment failed to induce G_2_/M arrest in HGC-27R cells (Figs. 2a, b), similarly, nocodazole’s impact on G_2_/M phase ratio in HGC-27R cells was inferior to in HGC-27P cells (Figs. 2a, b). These data were validated by an upregulation of cyclin B1 in HGC-27R cells (Fig. 2c), suggesting disrupted mitosis in PTX-resistant GC cells. We then evaluated the responses of mitotic molecules to PTX and nocodazole. Treatment of PTX or nocodazole induced significant G_2_/M phase arrest, i.e., upregulated cyclin B1/pHH3 and downregulated pCDK1 in parental cells, whereas these changes were merely observed in PTX-resistant cells (Fig. 2d). Thus, HGC-27R cells displayed an insensitivity to antimitotic agents and subsequent cell cycle inhibition, which might be due to its acquisition of PTX resistance.

### PTX-resistant GC cells possessed greater anti-apoptotic capacity than parental cells

**Fig. 3:**
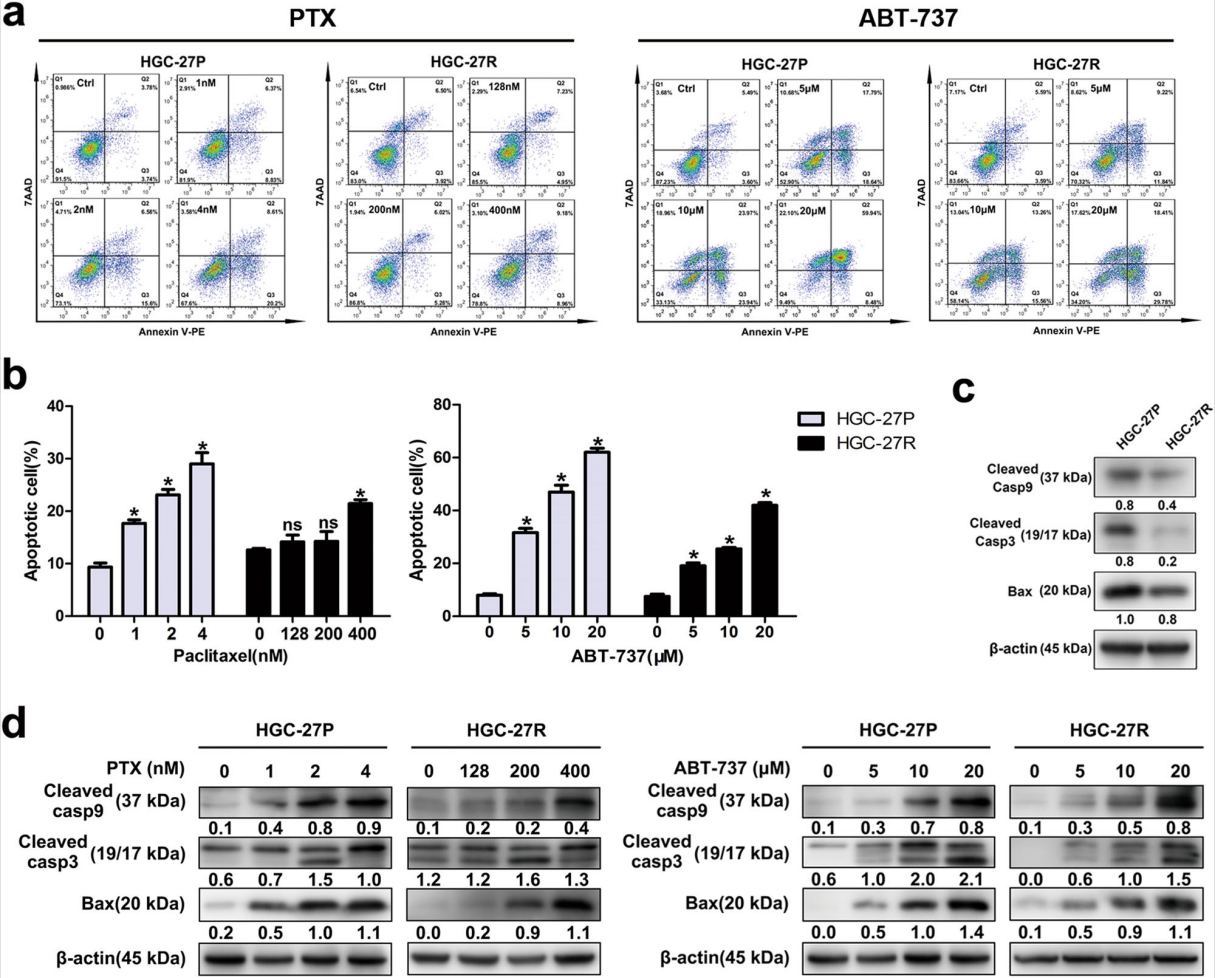
PTX-resistant GC cells possessed greater anti-apoptotic capacity than parental cells. **a**, **b** Parental and PTX-resistant GC cells double stained with Annexin V-PE/7-AAD and analyzed by flow cytometry after PTX treatments for 48 h. **c**, **d** Pro-apoptotic protein expression measured using western blot. Data expressed as mean ± S.D. of three independent experiments. *p < 0.05

PTX-mediated mitotic arrest causes apoptosis;^24^ however, resistance to apoptosis frequently arises during long-term treatment. So, we compared the anti-apoptotic capacity of PTX-resistant and parental cells with induction of both PTX and ABT-737, a widely used pro-apoptotic drug. Higher doses of PTX were required to induce apoptosis in HGC-27R cells than the progenitors (Figs. 3a, b), whereas HGC-27R cells were also less sensitive to ABT-737-induced apoptosis than HGC-27P cells (Figs. 3a, b). Molecular investigations indicated a decline in pro-apoptotic proteins (caspase-9, caspase-3, and Bax) in PTX-resistant cells (Fig. 3c). When treated with increased gradients, both PTX and ABT-737 evidently induced expression of pro-apoptotic proteins (caspase-9, caspase-3, and Bax) in progenitor cells, yet this induction was less effective in HGC-27R cells (Fig. 3d). Taken together, PTX-resistant GC cells displayed a greater anti-apoptotic capacity than parental cells.

### Targeting the PI3K/Akt/mTOR pathway with BEZ235 yielded strong antitumor effects in PTX-resistant GC cells

**Fig. 4:**
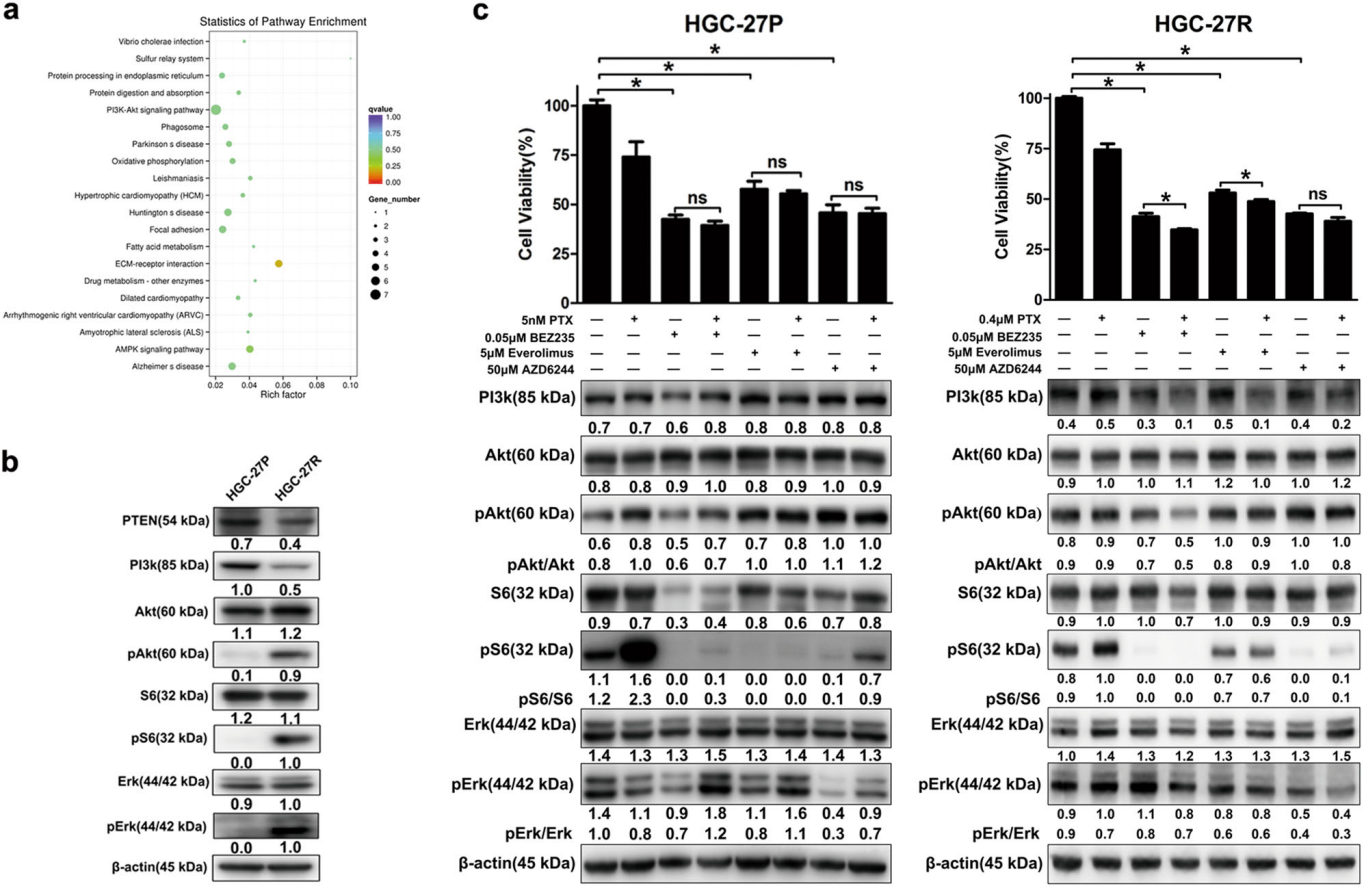
Targeting the PI3K/Akt/mTOR pathway with BEZ235 yielded best antitumor effects in PTX-resistant GC cells. **a** Significantly enriched pathways in PTX-resistant HGC-27R cells compared with parental cells identified through KEEG analysis. **b** Western blot quantification of protein expression in parental and PTX-resistant GC cells. **c** Cell viability and protein expression of parental cells and their resistant clones assayed by CCK-8 assay and western blot, respectively. Data expressed as mean ± S.D. of three independent experiments. *p < 0.05

To identify possible molecule candidates for treating PTX-resistant GC, we performed RNA-seq on HGC-27P and HGC-27R cells. Figure 4a shows an enrichment of the PI3K/Akt/mTOR pathway in PTX-resistant cells according to Kyoto Encyclopedia of Genes and Genomes (KEGG) pathway enrichment analysis. We studied changes in cross-talk between PI3K/Akt/mTOR and MAPK pathways, and PTX-resistant cells had PI3K/Akt/mTOR pathway activation marked by decreased PTEN and increased Akt and S6 phosphorylation along with activated MAPK signaling marked by enhanced Erk phosphorylation (Fig. 4b). Thus, PTX resistance in GC may be due to activation of PI3K/Akt/mTOR and MAPK signaling.

To understand the therapeutic potential of targeting these two pathways to treat PTX-resistant GC, we assessed the antitumor effects of PI3k/mTOR dual inhibitor BEZ235, mTOR inhibitor everolimus, and MEK inhibitor AZD6244 in paired HGC-27 cells. All three drugs alone or in combination with PTX suppressed cell growth and S6 and Erk activity in PTX-resistant cells, and BZE235 also inhibited Akt activity, suggesting that targeting the PI3K/Akt/mTOR pathway or MAPK pathway, especially dual PI3K/mTOR inhibition, may be valid for PTX-resistant cells (Fig. 4c). All three drugs (BEZ235, Everolimus, and AZD6244) alone suppressed cell growth and S6/Erk activity in both HGC-27R and HGC-27P cells. However, Akt re-activation was observed in parental cells treated with Everolimus or AZD6244. When combined with PTX, these drugs elicited higher inhibition on cell viability and Erk activity in resistant cells, yet in parental cells slightly augmented Erk activity (Fig. 4c). These might be due to that targeting PI3K/Akt/mTOR or MAPK pathways would induce re-activation of Akt/Erk in progenitor cells, whereas after the acquisition of PTX resistance, this feedback was attenuated, making HGC-27R more sensitive to combined agents. This might be a promising instruction to overcome induced chemoresistance in cancer treatment, yet our hypothesis required further investigation.

### BEZ235 had antitumor effects partly through the PI3K/Akt/mTOR pathway in PTX-resistant GC

**Fig. 5:**
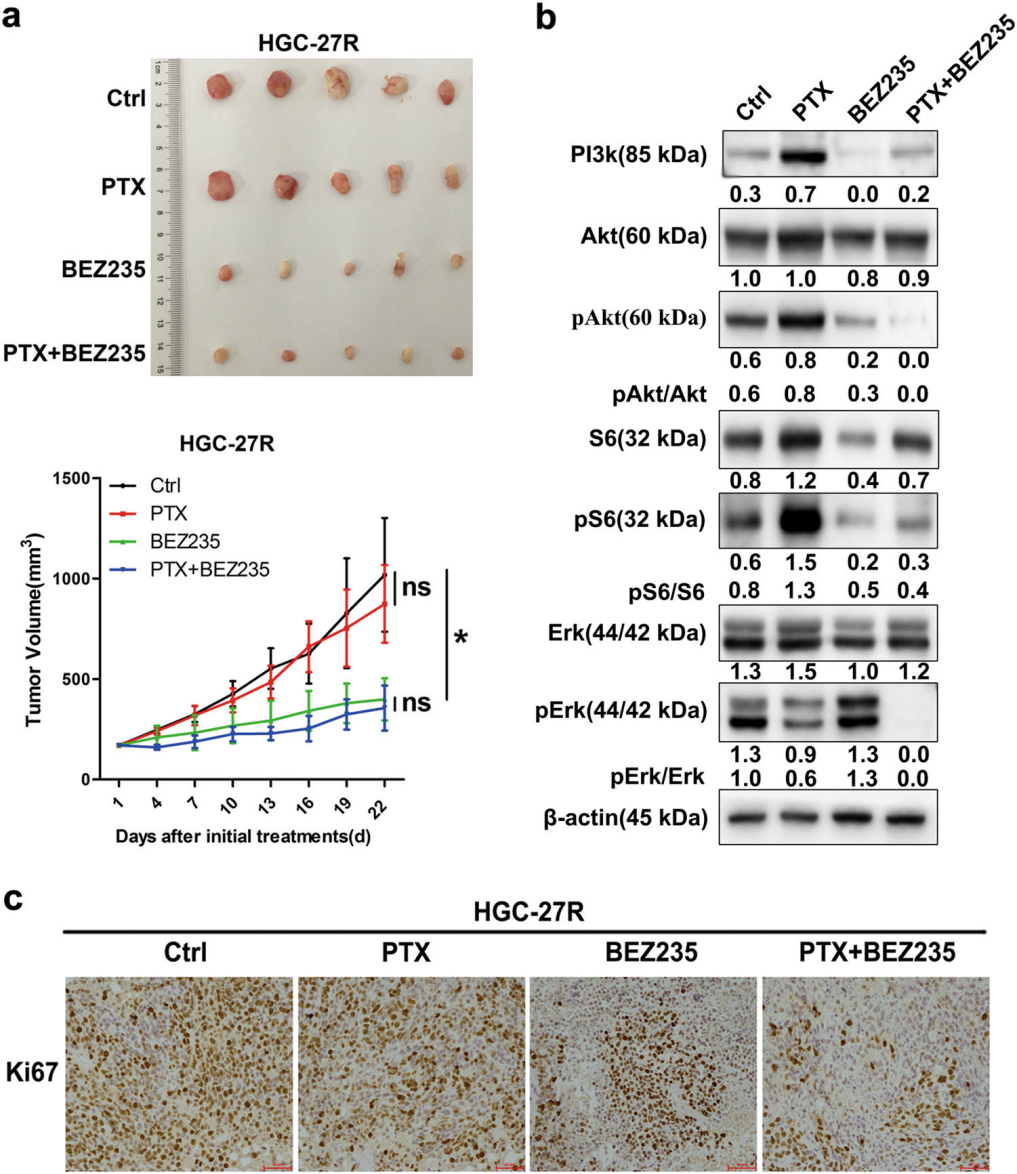
BEZ235 had antitumor effects partly through the PI3K/Akt pathway in PTX-resistant GC. **a** BEZ235 (45 mg/kg/day, daily, by gavage) with or without PTX (10 mg/kg/week, weekly, ip) given to mice bearing HGC-27R tumors for 21 days. Tumor volume expressed as mean ± S.D. (*n* = 5 per group). **c** FFPE sections stained with Ki-67 for IHC analysis. Original magnification, ×200. **b** Tumor lysates immunoblotted for corresponding proteins

BEZ235 yielded the best response in PTX-resistant GC in vitro. Figure 5a shows that BEZ235 alone or combined with PTX suppressed tumor growth in PTX-resistant xenografts derived from HGC-27R cells. BEZ235 attenuated Akt and S6 activity and decreased proliferation indicated by Ki-67 in HGC-27R-derived tumors (Figs. 5b, c). Thus, BEZ235 achieved antitumor activity at least partially by suppressing the PI3K/Akt/mTOR pathway in PTX-resistant GC.

## Discussion

PTX is a front-line agent widely used in antitumor chemotherapies^25^. However, despite its potent and extensive antitumor activity, the efficacy of PTX is limited by resistance that inevitably is acquired after long-term exposure. To date, several mechanisms underlying PTX resistance in tumors have been reported, such as increased drug efflux, tubulin mutation, altered microtubule dynamics, and impaired cell death signaling^13^. Treating PTX-resistant cancers has been studied using microtubule-targeting agents, CDK1, histone deacetylase (HDAC) or autophagy inhibitor, or apoptosis modulators, or by reducing drug efflux^14,17–19,26,27^. However, except for fibroblast growth factor receptor blockade^20^, therapeutic strategies for PTX-resistant GC are uninvestigated. Thus, we attempted to identify potential molecular candidates and to develop therapeutic options for PTX-resistant GC.

We established PTX-resistant GC cell sublines and integrated biological traits and molecular mechanisms of PTX resistance. PTX is a mitotic inhibitor that stabilizes microtubules by binding to β-tubulin, thereby blocking progression at the G_2_/M phase and inducing apoptosis^24^. Presumably, acquired resistance of GC to PTX may be conferred by microtubular disorder, reduced responses to antimitotic drugs, and apoptotic resistance. Thus, PTX-resistant GC cells in our study had disorganized overexpressed β-tubulin III, attenuated cell cycle changes in response to antimitotic agents, and greater anti-apoptotic capacity (Figs. 1–3). We previously validated the predictive values of β-tubulin III in PTX sensitivity in GC^6^. Being a chief target of PTX, β-tubulin III has a role in primary and acquired resistance to PTX, and this finding has been reported in other PTX-resistant cancers, such as lung, ovarian, and prostate cancer^9,12,28,29^. However, this study suggest modest upregulation of β-tubulin III after acquisition of resistance, implying that other molecules may be involved in PTX resistance in GC. For instance, cytoskeleton-associated proteins, such as other tubulin family isotypes, microtubule-associated proteins, and actin skeletons (especially γ-actin), have been determined to be associated with PTX resistance in tumors^13^, whereas our RNA-seq data also identified an upregulation of ACTG1 (encoding the protein γ-actin) in HGC-27R (Table S1). Thus, the roles of other cytoskeleton-associated molecules in PTX resistance of GC require an investigation.

Alternations of G_2_/M regulators contribute to PTX resistance in cancers. Cyclin B1 is a crucial regulator in G_2_/M transition, whose upregulation has been identified to confer resistance to PTX in tumors^30,31^. Consistently, increased cyclin B1 was observed in our established PTX-resistant cells (Fig. 2c). To further evaluate functional status of G_2_/M regulators, we also compared response of parental cells and their resistant clones with two antimitotic drugs, PTX and nocodazole. By targeting microtubules, these agents result in cyclin B1/pHH3 accumulation and CDK1 de-phosphorylation at Y15^30–33^, thus mediating G_2_/M block. According to our results, PTX-resistant cells had reduced responses to PTX or nocodazole, giving a hint that the acquisition of PTX resistance attenuated PTX-mediated inability of mitotic spindle formation.

Furthermore, apoptosis triggered by mitotic arrest is a major route for PTX-induced antitumor effects^34^. PTX activates caspase-dependent apoptotic pathways after inducing G_2_/M arrest in prostate cancer cells^35^. Given that mitosis stalling can release the apoptotic brake, we then tested whether apoptotic resistance occurred in PTX-resistant GC cells. Indeed, PTX-resistant cells had greater anti-apoptotic capacity, as indicated by reduced caspase-9, caspase-3 and Bax (Fig. 3c). Reduced Bax abundance also has been observed in other PTX-resistant cancers^12,14^. Apoptotic induction, such as targeting HDAC, has been considered as a candidate to treat PTX-resistant cancers^19^. Nevertheless, as we found that impact of PTX and ABT-737 on these pro-apoptotic proteins was less effective in resistant clones than in progenitors (Fig. 3d), targeting PI3K/Akt/mTOR might be a more efficient option against PTX-resistant cancer than apoptotic induction.

EMT participates in resistance to chemotherapy in numerous cancers. Hallmarks of EMT include loss of expression and function of epithelial markers such as E-cadherin, cytokeratin, and claudin, as well as concomitant increases in the abundance of mesenchymal markers, such as vimentin and N-cadherin^36^. The EMT phenotype has been linked to upregulation of β-tubulin III in PTX-resistant cancer cells^12^. Our PTX-resistant GC cells experienced an EMT process featuring elevated vimentin and reduced keratin 20 along with higher invasion and migration capacity (Figs. 1d, e and Table S1). Also, our RNA-seq data revealed the enrichment of the extracellular matrix pathway and changes in other EMT-related genes in PTX-resistant HGC-27R cells, including CLDN12, SPARC, and COL1A1 (Fig. 4a and Table S1). More research is needed to validate expression of these molecules and ascertain the acquisition of an EMT phenotype in PTX-resistant GC cells.

Activated PI3K/Akt/mTOR or MAPK pathways have been documented in PTX-resistant prostate and breast cancers^21,37^, and this occurred in our PTX-resistant GC cells (Fig. 4b). However, PI3K abundance decreased in HCG-27R cells, and this was in disagreement with the work of Jeong’s group^38^. This difference may be explained by feedback repression by activated downstream S6^39^. However, the therapeutic potential of targeting the PI3K/Akt/mTOR or MAPK pathway for PTX-resistant GC is uncertain. Several drugs targeting nodes of the two pathways have been developed, and three representative agents (PI3k/mTOR dual inhibitor BEZ235, mTORC1 inhibitor everolimus, and MEK inhibitor AZD6244) FDA-approved for the treatment of various cancers were used in our work. As reported, BEZ235 inhibits multiple class I PI3K isoforms and mTORC1/2 kinase activity, exerting potent anticancer activity, and attenuating PI3k re-activation and mTORC2-mediated Akt re-activation^39,40^. Conversely, everolimus is a FDA-approved rapamycin analog, which only targets mTORC1 kinase with a susceptibility of activating feedback loops^39,40^. AZD6244 is a potent and selective mitogen-activated extracellular signal-related kinase kinase 1/2 (MEK1/2), also called Erk kinase inhibitors that effectively inhibits phosphorylation of extracellular signal-regulated kinase 1/2 (Erk1/2), substrates of Mek1/2^41^. We found that all three drugs (BEZ235, everolimus, and AZD6244) alone or in combination with PTX reduced cell growth by inhibiting S6 and Erk activity in PTX-resistant cells and that BEZ235 was most efficacious (Figs. 4c, 5). Unlike everolimus, BEZ235 also lowered Akt activity in HGC-27R cells. Better antitumor activity with BEZ235 than mTOR inhibitors alone or in combination with PTX also has been reported in cancers^42,43^. The superiority of BEZ235 over mTOR inhibitors can be due to its dual blockade on upstream and downstream of Akt, which avoids Akt feedback re-activation induced by mTOR inhibitors^40^. These data suggest that targeting the PI3K/Akt/mTOR pathway and MAPK pathway, especially with a PI3K/mTOR dual inhibitor, may be efficient to treat PTX-resistant GC. Moreover, all three drugs regulated PI3K/Akt/mTOR and MAPK pathways in this study, and any off-target effects could be caused by overlapping regulation of these two signaling pathways. As reported, PI3K can positively regulate the MAPK pathway, whereas Akt and its downstream effectors negatively regulate the pathway in a content-dependent manner^44^. Interestingly, although parental cells responsed to all three drugs, they displayed a slight activation of Akt/Erk (Fig. 4c). These data indicated that PI3K/Akt/mTOR and MAPK pathways were activated during the acquisition of PTX resistance, yet their ability to re-activate as a feedback of targeted inhibition was attenuated. This might be a promising instruction to overcome induced chemoresistance in cancer treatment, yet our hypothesis required a further investigation.

Notably, in vivo data revealed that PTX alone activated PI3k/Akt/mTOR pathways, which was consistent with activation of PI3k/Akt/mTOR signaling during PTX resistance acquisition. Thus, further PTX stimulation in PTX-resistant-cells might serve as a pro-survival factor, which evoked PI3K/Akt/mTOR signaling and cascade responses. Conversely, combination of BEZ235 and PTX elicited the best antitumor effect in PTX-resistant GC with remarkable inhibition of pAkt, pS6 and pErk (Fig. 5). This might be due to that PI3k/Akt/mTOR signaling was activated after PTX monotherapy as a feedback response, yet was blocked by BEZ235 after PTX-BEZ235 dual administration. Thus, the feedback upregulation of Akt by PTX was abrogated after PTX-BEZ235 dual treatment, which in turn augmented BEZ325 and PTX’s antitumor activity. However, our hypothesis remains to be validated.

### Conclusions

PTX-resistant GC cells was characterized by microtubular disorder, an EMT phenotype, reduced responses to antimitotic drugs, and apoptotic resistance. Targeting the PI3K/Akt/mTOR or MAPK pathway, especially with PI3K/mTOR dual inhibition, offered antitumor potentials in PTX-resistant GC, so this may represent a novel treatment strategy for PTX-resistant GC. The underlying mechanisms of the effects and any clinical applications merit further investigation.

## Materials and methods

### Reagents and antibodies

BEZ235 (Dactolisib), AZD6244 (Selumetinib), Everolimus (RAD001), nocodazole, and ABT-737 were purchased from Selleck Chemicals (Houston, TX). PTX was purchased from Beijing Union Pharm (Beijing, China). Reagents were formulated and stored until use according to the manufacturer’s protocols. Most antibodies were purchased from Cell Signal Technology (CST, Danvers, MA), including primary antibodies against Keratin 20 (#13063), N-cadherin (#13116), vimentin (#5741), PI3K (#4257), Akt (#4691), pAkt (#4060), S6 (#2217), pS6 (#4858), PTEN (#9188), Erk (#4695), pErk (#4370), cyclin B1 (#12231), CDK1 (#28439), pCDK1 (#4539), pHH3 (#53348), cleaved caspase-9 (#7237), cleaved caspase-3 (#9664), and Bax (#5023) and secondary horse radish peroxidase (HRP)-conjugated goat anti-rabbit and anti-mouse antibodies. Antibodies against β-tubulin III (#Ab68193), Ki-67(#ZM-0167), and β-actin (#014M4759) were purchased from Abcam (Cambridge, UK), ZSJB-BIO (Beijing, China), and Sigma-Aldrich (St. Louis, MO), respectively.

### Cell lines and cell culture

We purchased the HGC-27 human GC cell line from the Cell Bank of Chinese Academy of Sciences (Beijing, China). Cell lines were cultured in RPMI-1640 (Gibco BRL, Gaithersburg, MD), supplemented with 10% fetal bovine serum (Gibco BRL) and 1% penicillin–streptomycin (HyClone, Logan, UT), and incubated at 37 °C in a humidified atmosphere with 5% CO_2_.

### Cell viability assay

We plated cells (3000–5000 per well) onto 96-well plates and incubated overnight in complete medium. After indicated drug exposures for 48 h, cell viability was measured using a CCK-8 kit (Dojindo Laboratories, Japan) according to the manufacturer’s instructions. Absorbance was measured at 450 nm using a spectrophotometer. IC_50_ was calculated by Graphpad Prism Version 5.0 software. The resistant index (RI) was calculated utilizing the following formula: RI = IC_50_ of resistant cells/IC_50_ of parental cells.

### Immunofluorescent staining

Cells (300,000 cells/ml) were seeded on a 35 mm glass bottom dish (NEST, Jiangsu, China) and incubated overnight in complete medium. After drug treatments for 12 h, we fixed cells with 4% paraformaldehyde (Solarbio, Beijing, China) for 10 min, permeabilized with 0.5% Triton X-100 (Amresco, Solon, OH) for 20 min, and blocked with 5% bull serum albumin (Amresco) for 30 min at room temperature. Cells were probed with primary antibody against β-tubulin III (1:50) at 4 °C overnight and Alexa Fluor 488-conjugated goat anti-rabbit IgG (Molecular Probes, Eugene, OR, 1:500) in the dark for 1 h at room temperature. We counterstained cell nuclei with diamidino-phenyl-indole (DAPI) (Beyotime, 1:3000) in the dark for 5 min at room temperature. All reagents were diluted in phosphate-buffered saline (PBS) and all steps were followed by 5-min PBS washing three times. We captured images with ZEN version 2012 software (Zeiss, Gottingen, Germany) using a laser scanning confocal microscope LSM 780 (Zeiss). We applied the same exposure settings for all images of identical proteins.

### Western blot

We lysed the cells using a CytoBuster protein extraction reagent (Merck Millipore, Darmstadt, Germany) in the presence of protease and phosphatase inhibitor cocktail tablets (Roche, Basel, Switzerland). We measured protein concentration using a BCA Protein Assay Kit (Beyotime, Jiangsu, China). Soluble lysates containing about 20 μg proteins per sample were resolved with sodium dodecyl sulfate–polyacrylamide gel electrophoresis and transferred to a polyvinylidene fluoride membrane (Merck Millipore). After blocking using 5% bull serum albumin or fat-free milk, membranes were probed with primary antibodies (dilutions were 1:1000 except for β-actin at 1:10,000) at 4 °C overnight and secondary antibodies (1:2000) at room temperature for 1 h. We visualized the signals using an Amersham Imager 600 (GE Healthcare, UK) after incubation with clarity Western ECL substrate (Bio-Rad, Hercules, CA). We quantified protein expression using Image J Version 1.48 software (NIH, Bethesda, MD) and used β-actin as a loading control. Relative expression of indicated proteins were normalized to β-actin.

### Cell invasion and migration assays

Cells were seeded in the upper chambers of 24-well Transwell plates with/without pre-coated Matrigel (Corning, New York, NY) following the manufacturer’s instructions for invasion/migration assays, respectively. The lower chambers were filled with culture medium supplemented with 10 % FBS. Invaded and migrated cells (after incubation for 48 and 24 h, respectively) in the lower chambers were fixed and stained with crystal violet and counted under a microscope.

### Cell cycle assay

After drug interventions for 12 h, cells were collected, washed with PBS, and fixed in 70% immediately prepared precooled ethanol overnight at 4 °C. After washing with PBS three times, cells were stained with propidium iodide (BD Biosciences, Erembodegem, Belgium) at room temperature for 15 min in the dark according to the instructions, followed by flow cytometry analysis within 1 h (BD Biosciences). The distribution of cells at specific cell cycle stages was assessed with ModFit Version 3.0 software (Verity Software House, Topsham, ME).

### Apoptosis assay

After drug treatment for 48 h, cells were double-stained with Annexin V-Phycoerythrin (PE) and 7-amino-actinomycin (7-AAD) (BD Biosciences) at room temperature in the dark as described in the vendor’s protocol, followed by flow cytometry analysis within 1 h (BD Biosciences). We analyzed proportions of apoptotic cells using FlowJo Version 7.6.1 software (FlowJo, Ashland, OR).

### RNA-seq

We prepared total RNA using the Trizol method and confirmed RNA integrity using an Agilent 2100 Bioanalyzer (Agilent Technologies). We performed next-generation sequencing using an Illumina HiSeq instrument according to the manufacturer’s instructions (Illumina, San Diego, CA). Sequences were processed and analyzed by Novogene (Beijing, China).

### Animal experiments

We suspended HGC-27R cells in PBS at a concentration of 2 × 10^7^/mL and subcutaneously injected a 100 µL cell suspension into the dorsal right flank of 5-week-old female Balb/c nude mice (Vital River Laboratories, Beijing, China). When tumor volume reached ~ 100 mm^3^, we randomized mice into four groups (*n* = 5 per group): (1) control: 100 μL PBS daily (ip); (2) PTX: PTX (10 mg/kg weekly, ip); (3) BEZ235: BEZ235 (45 mg/kg daily, by gavage); (4) combination: PTX (10 mg/kg weekly, ip) plus BEZ235 (45 mg/kg daily, by gavage). We treated animals for 3 weeks and measured tumor size and body weight every 3 days. Tumor volume was calculated using the formula V = L × W^2^ × 1/2 (V, volume; L, length of tumor; W, width of tumor). After final drug administrations, mice were killed, tumors were stripped, samples were processed for formalin-fixed and paraffin-embedded (FFPE) sections or western blot. All animal experiments were approved by Peking University Cancer Hospital’s Institutional Animal Care and Use Committee and complied with the internationally recognized Animal Research: Reporting of In vivo Experiments guideline.

### Immunohistochemistry

We deparaffinized, hydrated, and retrieved the tumors sections and removed endogenous peroxidase. Then, samples were blocked with 5% bovine serum albumin according to standard procedures, and 4-μm thick FFPE sections were incubated with primary antibody against Ki-67 (1:300) overnight at 4 °C followed by IgG/HRP polymer (ZSGB-BIO) and diaminobenzidine substrate (Gene Tech, Shanghai, China). Staining results were independently evaluated by two pathologists from the Department of Pathology in Peking University Cancer Hospital as described in our previous study^45^.

### Statistical analysis

We performed statistical analysis using the SPSS Version 21.0 software. We analyzed differences between groups using a Student’s *t-*test, and we assessed one-way or repeated measures with analysis of variance (*p* < 0.05 was considered statistically significant).

## Supplementary information

Table S1

Figure S1

Supplementary material Figure legend
