# Supplementary material for: Dual PI3K/mTOR inhibitor BEZ235 as a promising therapeutic strategy against paclitaxel-resistant gastric cancer via targeting PI3K/Akt/mTOR pathway
Source: Cell Death Dis. 2018 Jan 26;9(2):123. doi: 10.1038/s41419-017-0132-2 (PMC5833539; doi:10.1038/s41419-017-0132-2)
Supplement: Supplementary file 1 — Table S1 [file 41419_2017_132_MOESM1_ESM.docx]

**Table S1** Differentially expressed genes of EMT and microtubule-associated proteins between HGC-27P and HGC-27R cells identified by RNA-seq

| Gene ID | Associated Gene Name | Readcount of  HGC-27R | Readcount of  HGC-27P | Fold change  (log 2) | *P* value |
| --- | --- | --- | --- | --- | --- |
| ENSG00000026025 | VIM | 1043.1 | 376.6 | 1.5 | 1.66E-50 |
| ENSG00000184009 | ACTG1 | 977.9 | 355.4 | 1.5 | 6.87E-47 |
| ENSG00000157224 | CLDN12 | 26.8 | 216.6 | -3.0 | 1.23E-44 |
| ENSG00000113140 | SPARC | 128.0 | 30.6 | 2.1 | 2.05E-12 |
| ENSG00000108821 | COL1A1 | 1422.9 | 567.7 | 1.3 | 4.62E-56 |
