# Supplementary figures and images for: Dual PI3K/mTOR inhibitor BEZ235 as a promising therapeutic strategy against paclitaxel-resistant gastric cancer via targeting PI3K/Akt/mTOR pathway
Source: Cell Death Dis. 2018 Jan 26;9(2):123. doi: 10.1038/s41419-017-0132-2 (PMC5833539; doi:10.1038/s41419-017-0132-2)

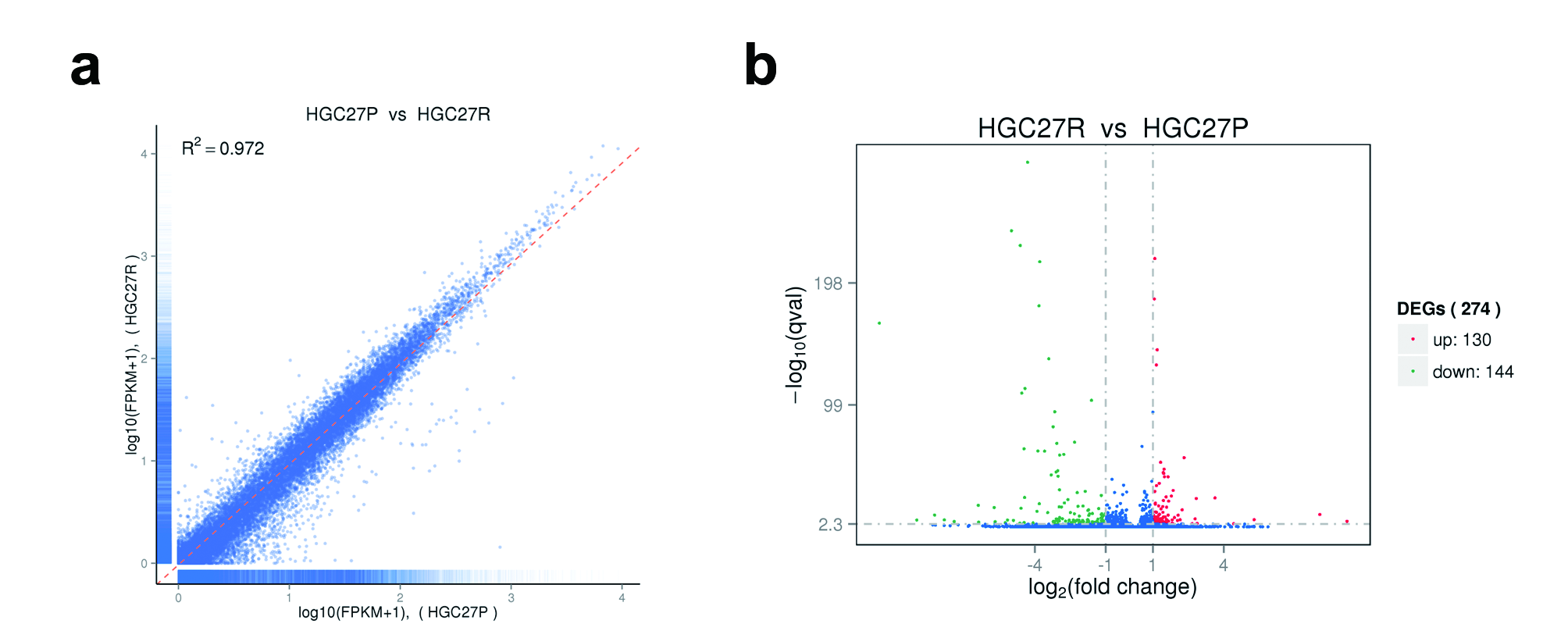

Supplement: Supplementary file 2 — Figure S1 [file 41419_2017_132_MOESM2_ESM.tif]
