# Supplementary material for: Dual PI3K/mTOR inhibitor BEZ235 as a promising therapeutic strategy against paclitaxel-resistant gastric cancer via targeting PI3K/Akt/mTOR pathway
Source: Cell Death Dis. 2018 Jan 26;9(2):123. doi: 10.1038/s41419-017-0132-2 (PMC5833539; doi:10.1038/s41419-017-0132-2)
Supplement: Supplementary file 3 — Supplementary material Figure legend [file 41419_2017_132_MOESM3_ESM.docx]

**Supplementary material 1: Table S1.** Differentially expressed genes of EMT and microtubule-associated proteins between HGC-27P and HGC-27R cells identified by RNA-seq.

**Supplementary material 2: Figure S1.** Correlation and differentially expressed genes (DEGs) between HGC-27P and HGC-27R cells**.** (**a**) Scatter plot of Pearson correlation between samples. Correlation coefficient between HGC-27P and HGC-27R cells was 0.972, suggesting two cell lines were derived from one cell line. (**b**) Volcano plot of differentially expressed genes between HGC-27P and HGC-27R cells (DEGs screened). Red points represent 130 upregulated genes of statistical significance in HGC-27R cells; green points represent 144 downregulated genes.
